# Molecular spectrum of secretome regulates the relative hepatogenic potential of mesenchymal stem cells from bone marrow and dental tissue

**DOI:** 10.1038/s41598-017-14358-0

**Published:** 2017-11-08

**Authors:** Ajay Kumar, Vinod Kumar, Vidya Rattan, Vivekananda Jha, Arnab Pal, Shalmoli Bhattacharyya

**Affiliations:** 1 Department of Biophysics, PGIMER, Chandigarh, India; 2 Department of Nephrology, PGIMER, Chandigarh, India; 3 Unit of Oral and Maxillofacial surgery, Oral health science centre, PGIMER, Chandigarh, India; 4 Department of Biochemistry, PGIMER, Chandigarh, India; 5 Present Address: University of Oxford, Oxford, UK

## Abstract

Liver regeneration is a spontaneous process that occurs after liver injury, but acute liver failure is a complex and fatal disease which is difficult to treat. Cell-based therapies are promising alternative therapeutic approach for liver failure and different cell sources have been tested in this regard. We investigated the comparative hepatogenic potential of human bone marrow stem cells (BMSC) with stem cells derived from human dental pulp (DPSC), apical papilla (SCAP) and follicle (DFSC) during this study. Hepatogenic potential of stem cells was assessed by functional assays at both genetic and protein level. We observed higher expression of most of the hepatic markers post differentiation in DPSCs compared to other cell types. LC-MS/MS analysis of stem cell secretome revealed the presence of different proteins related to hepatogenic lineage like growth arrest specific protein 6, oncostatin M, hepatocyte growth factor receptor etc. Interactome and Reactome pathway analysis revealed the interaction of DPSC/SCAP secretome proteins and these proteins were found to be associated with various pathways involved in lipid transport and metabolism. To the best of our knowledge, this is the first study regarding detailed investigation of hepatogenic potential of BMSCs v/s DMSCs (DPSC, SCAP & DFSC) along-with secretome characterization.

## Introduction

Liver transplantation is the only therapeutic option for many congenital and acquired liver diseases. The widespread application of liver transplantation is limited by a paucity of liver donors, risk of surgical complications, graft-versus-host disease, and high medical costs. There is a need for development of alternative methods of treatment and regenerative medicine offers a novel approach for treatment of liver disease. Currently, cell therapy and tissue/organ engineering are the main regenerative medicine techniques. Cell therapy is less expensive and invasive compared to organ transplantation or tissue engineering. Liver regeneration can be stimulated by cell therapy with hepatocytes, hematopoietic stem cells, or mesenchymal stem cells (MSCs). Mesenchymal stem cells work either by providing trophic support factors at the site of injury or by differentiation of some of the stem cells into hepatocytes. Although there has been a significant improvement in differentiation protocols to improve the efficacy and functionality of hepatocyte differentiation^1,2^ further refinement in hepatic differentiation protocols are needed to make their application more feasible in clinical settings. Defining a suitable source of stem cells for obtaining functional hepatocytes is also crucial for development of effective liver regeneration therapy. Functional hepatocytes have been successfully derived from various types of stem cells like embryonic stem cells (ESCs)^3^, induced pluripotent stem cells (iPSCs)^4^, bone marrow stem cells (BMSCs)^5^, adipose derived stem cells (ADSCs)^6^, umbilical cord derived stem cells (UC-MSCs)^7^ etc. Earlier, Khanjani *et* *al*. reported that menstrual blood derived stem cells (MnSCs) had higher hepatic differentiation potential compared to BMSCs. This conclusion was based on higher expression of hepatocyte markers in differentiated MnSCs^8^. A comparative analysis of hepatogenic potential between placenta derived stem cells (PDSC), ADSC, & BMSC showed PDSC having higher tendency to form functional hepatocytes based on hepatic gene expression and other parameters like urea production, elevated expression of albumin (ALB) and various cytokines^9^. However, in another study, Visconti *et* *al*. found no significant difference in the potential of ADSC & BMSC to differentiate into hepatocytes^10^. Apart from stem cells derived from different sources, difference in hepatic differentiation potential of stem cells from different donors has also been reported. A study by Kajiwara and colleagues shed light on donor-dependent variations in for hepatic differentiation in various clones of human induced pluripotent stem cells (hiPSCs). This study established that origin of stem cells but not the derivation method is an important contributor for hepatic differentiation of stem cells.

Derivation of terminally differentiated cells for different diseases is important in the field of regenerative medicine, so there is a continuous search for specific cells suitable for efficient differentiation into a committed lineage. Dental tissue derived stem cells offer an exciting area of research to explore new avenues in stem cell therapy. These cells are sturdy and versatile in nature^11^. Although hepatic differentiation of dental pulp stem cells has been reported before^12^ but a clear comparison between the efficacy of hepatic differentiation between bone marrow and dental stem cells (DPSC, SCAP and DFSC) has not been reported so far. Micro-environmental cues like biological scaffolds or paracrine factors can determine the differentiation ability of stem cells. Wang *et* *al*. have demonstrated the role of biological matrix in directing iPSC differentiation to osteoblasts which can be highly beneficial for tissue engineering purposes^13^. A numbers of evidences suggest that paracrine factors released by stem cells might act as major facilitators of the therapeutic action of stem cell and the concept of secretory molecules, collectively called as secretome is rapidly gaining importance in regenerative medicine^14–16^. The paracrine factors produced by adipose stem cells have been shown to increase the liver regeneration in mice through JAK/STAT signaling^17^. So we analyzed the proteomic profile of the secretome from these four stem cells populations for any hepatic lineage related proteins which can explain the hepato-protective role of secretome obtained from these cells. To the best of our knowledge this is the first report of comparative secretome analysis pertaining to hepatic lineage proteins from stem cells of different origins. Furthermore, we have also defined the signatures peaks of BMSCs and DMSCs on hepatic differentiation by Fourier transform Infrared spectroscopy (FTIR).

## Material and Methods

### Patient recruitment and tissue processing

Tissue was processed from five young donors (6–25 years age) visiting PGIMER OPD for tooth extraction or impacted third molar removal. All experimental protocols were approved by Ethics Review Committee of PGI (IEC No. 9195/PG-12 ITRG/2571-72) and carried out as per Helsinki declaration. Written informed consent was obtained from all patients or guardian if the age of patient was less than 15 years. Extracted teeth were disinfected and carried to the cell culture lab for further processing.

### Primary culture of BMSCs & DMSCs

Five tissue samples for each stem cell type were obtained in sterilized conditions. Extracted dental pulp from molar, apical papilla and follicle were processed and cultured in α-MEM media by explant culture method as reported before^11,18^. BMSCs were separated by ficoll density gradient. DMSCs and BMMSCs were cultured in 5% CO_2_ and a temperature of 37 °C in α-MEM, 10% FBS, 10ng/ml epidermal growth factor (EGF), 10 ng/ml platelet derived growth factor (PDGF), L-ascorbic acid, glutamine, penicillin/streptomycin, L-glutamine and supplemented with 10 μl/ml ITS (Insulin, human transferring and selenous acid).

### Identification of Stem cell markers

Cells were dissociated from culture surface by using trypsin (Sigma) and incubated with corresponding FITC/PE conjugated antibodies for stem cell markers CD-90, CD-105, CD-73, endothelial marker CD-34, and hematopoietic marker CD-45. Cells were analyzed atleast in triplicates by using BD FACS Canto flow cytometer. Unstained cells were used to normalize background fluorescence and were gated as population P1 while positive population was indicated in gate P2.

### Cell proliferation

5 × 10^3^ cells of each stem cell type were cultured in presence of complete culture media supplemented with 10% FBS for a duration of 48hrs. After 48 hrs; EZBlue dye was added (10% volume of media) and procedure was performed as per manufacturer’s instructions. Optical density was recorded at a measurement wavelength of 570 nm by taking 600 nm as reference wavelength (Tecan ELISA reader cum spectrophotometer).

### Assessment of multipotency of the BMSCs and DMSCs

Multilineage differentiation potential of BMSCs and DMSCs was assessed by differentiating them into cellular derivatives of ectoderm (neural cells), mesoderm (osteocytes and adipocytes) and endoderm (hepatocytes). The differentiation protocols were followed as reported previously^11,18^. Briefly, Osteogenic differentiation was done with supplementation of dexamethasone, β glycerophosphate and mono-potassium phosphate while insulin and indomethacin in combination with dexamethasone were used for adipogenic differentiation. Neural differentiation was achieved by adding B-27, N2, G5, EGF and bFGF supplements in neurobasal media for 40 days. Osteogenic and adipogenic differentiation were assessed by alizarin red S and Oil Red O stain while neural differentiation was confirmed by immunofluorescence staining for Neurofilament antibody. Hepatic differentiation was established by treating the cells with a differentiation medium, consisting of low glucose DMEM supplemented with 20 ng/ml hepatocyte growth factor (HGF), 2 ng/ml epidermal growth factor (EGF), 0.1 μM dexamethasone, and 50 mg/ml ITS + premix for 14 days, followed by maturation medium (DMEM supplemented with 20 ng/ml Oncostatin M, 0.5 μM dexamethasone and 50 mg/ml ITS + premix) for another 14 days. Parallel control studies were performed and medium was changed when required. Cell number was counted to confirm cell density.

### Real Time-PCR analysis

RNA was isolated at the end of differentiation protocol using TRIZOL™ reagent (Invitrogen). Primer probes were used to determine transcript levels in triplicate for housekeeping gene and different genes of interest viz. α-Fetoprotein, Tyrosine aminotransferase, Albumin, and expression was normalized by housekeeping gene β-Actin/GAPDH.

### Uptake of low-density lipoprotein

Hepatic differentiation was evaluated by using LDL functional hepatocyte assay kit (Abcam) and procedure was performed as per manufacturer’s instructions. A combination of LDL DyLight fluorochrome and LDL primary and secondary antibodies were used for detection of LDL uptake and expression of LDL receptor respectively. After differentiation, live cells were stained with LDL DyLight fluorochrome (1:100) and observed under fluorescence microscope (Nikon Eclipse TS100). Background fluorescence was normalized equally for all cell types by employing NIS- Elements D4.13.00 software package supplied with microscope and exposure settings were kept same for all cells. Cells were then fixed, permeabilized, put in blocking solution and incubated with antibodies (primary and secondary) as per manufacturer’s instructions. DAPI was used as nuclear stain and cells were then photographed in FITC/DAPI and PI excitation (488/350/536 nm respectively) and emission wavelengths (532/470/617 nm respectively) (EVOS LED microscope). Minor brightness corrections were done equally in whole of the fluorescent image after combining individual images from all four differentiated stem cells. Fluorescence attained by each cell as a result of antibody expression and LDL uptake was measured individually for each cell with the help of Image cytometer (Invitrogen-TALI Image based Cytometer). Cells were excited using FITC/PI wavelength under red/green fluorescence function provided with instrument software and various parameters like intensity values, percentage positivity of cells for red (LDL uptake-LDL Dylight fluorochrome) and green (LDL receptor-LDL antibody) were recorded for every cell. These values were then used to quantify the LDL uptake and distribution of LDL receptor in differentiated hepatocytes from BMSCs and DMSCs.

### Liver function test

Differentiated hepatocytes from all four stem cell sources were trypsinized and washed in phosphate buffer saline. Cells were sonicated and then centrifuged at 4,000 rpm for duration of five minutes to remove cell debris. These cell lysates were then run into Roche Modular P autoanalyzer and various parameters of liver function like AST (Aspartate aminotransferase), ALT (alanine aminotransferase) and ALP (alkaline phosphatase) were recorded according to manufacturer’s instructions to correlate with the hepatic differentiation potential of different stem cell types.

### FTIR analysis

Cells from all four stem cell types (control as well as differentiated hepatocytes) were trypsinized to obtain the cell pellet which was dried for night at a temperature of 156 °C. Dried pellets in powder form were added with equal amount of KBR (potassium bromide). The spectra were acquired using FTIR (PerkinElmer, Spectrum Two) by running atleast 52 scans per sample. The peaks thus obtained were searched against corresponding FTIR database.

### LC-MS/MS analysis of secretome for hepatic lineage proteins

For secretome harvesting, stem cells at 60–70% confluence were cultured in 75 cm^2^ culture flasks in the presence of αMEM alone (48 hrs) without any growth factors or FBS after repeated washings in PBS. Conditioned media obtained after culture was harvested in cold conditions, centrifuged for five minutes at 3,000 rpm, and filtered. Secretome was obtained between passages numbered 3^rd^–7^th^ and total protein was isolated by phenol chloroform method. The final protein profile was checked in SDS-PAGE. LC-MS/MS profiling of the secretome was done on WATERS NanoLC-SYNAPT G2 HDMS instrument. The criterion used for identifying true Protein Hits was the false discovery rate (FDR) parameter set as 4%. (PLGS inbuilt cut off for Protein Identification). Per protein at least 2 peptides were matched with sample. Additionally, at least 5 MSMS fragment ions were found in the Protein and at least 3 Fragment MSMS ions per peptides. Protein analysis was done using WATERS Protein Lynx Global Server (PLGS) v4.1 against the respective UNIPROT database. Bioinformatics analysis was performed using STRING software^19^. Proteins were classified using functional annotation and interaction of hepatic lineage proteins were observed in evidence view.

### Statistical analysis

All experiments were performed atleast in triplicates and were repeated three times. Data was reported as means ± SD. All statistical analyses were performed using one-way ANOVA and paired t test.

Detailed methodology and associated supporting data is available with the corresponding author on special request.

## Results

### Isolation and characterization of mesenchymal stem cells from dental tissue and bone marrow

**Figure 1:**
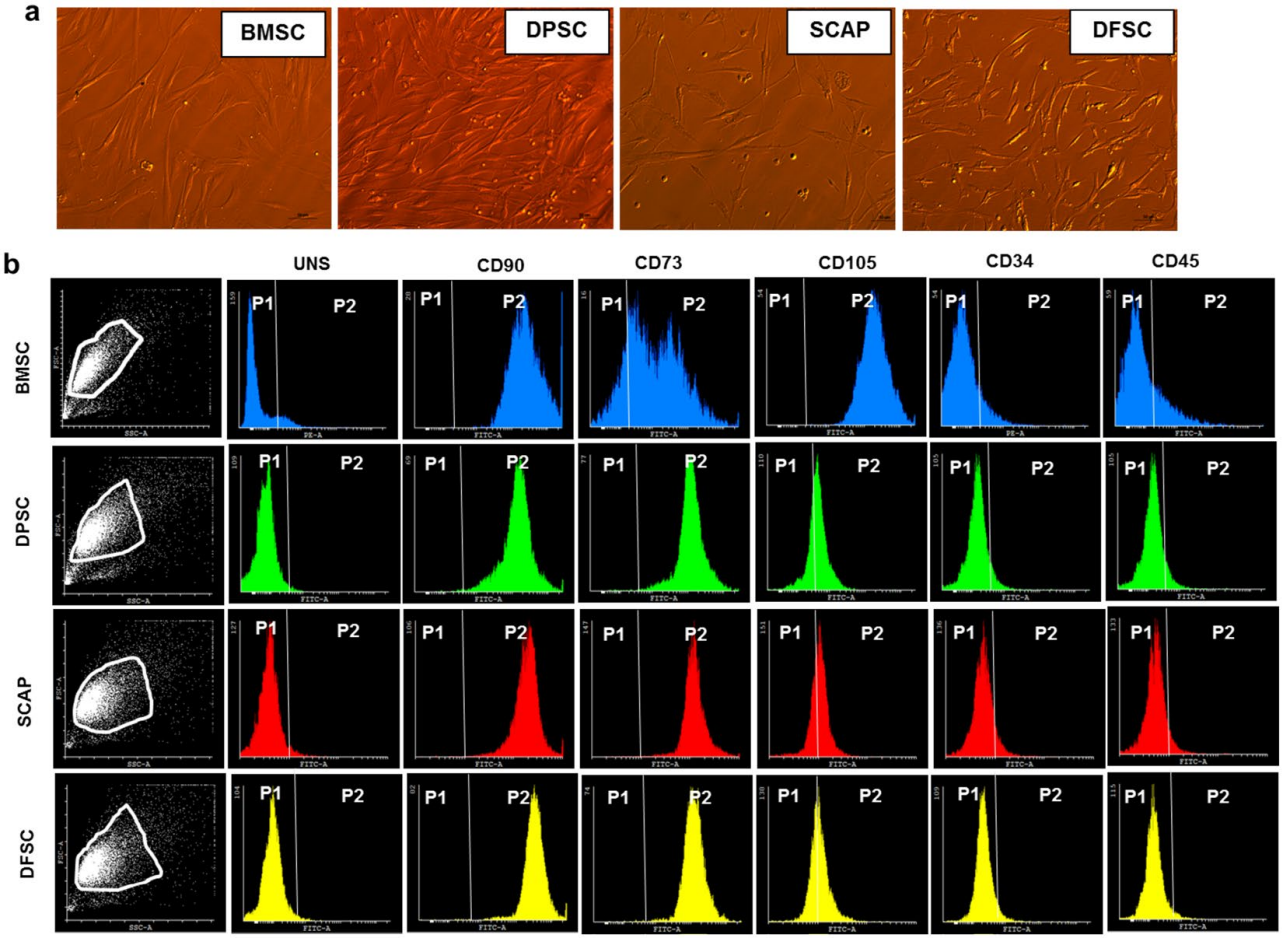
Establishment and characterization of primary culture of stem cells. (**a**) Differential Interference Contrast (DIC) images for BMSC, DPSC, SCAP and DFSC respectively, magnification-10X. (**b**) Flow cytometry characterization of BMSC, DPSC, SCAP and DFSC by different stemness antibodies (CD90,CD73 and CD105) and negative endothelial (CD34) and Hematopoietic (CD45) antibodies. Scale bar-50 µm.

DMSCs were obtained from tooth pulp (DPSC), papilla (SCAP) and follicle (DFSC) of extracted dental tissues (age 6–25 yrs). Impacted third molar was successfully removed by following proper procedure which involved screening of tooth by OPG, local anesthesia to the patient, incision in the area where tooth was to be removed. Incision flap was made to expose the tooth which was then cut and removed from the socket. Tissue was further transported to tissue culture laboratory for establishment of primary culture. It was observed that on the average, hDMSC migrated from the seeded tissues after 1–2 days of initial seeding. Thereafter, they formed small globule like cells which appeared in culture on 3^rd^–4^th^ day though migration rate was not uniform and in some tissues, cells migrated on 7^th^–8^th^ day. Different transitional stages were observed before formation of a confluent primary culture which involved initial bubble like stage with small rounded cells coming out of tissue. These cells initially formed a mesh- like network and subsequently achieved spindle shaped morphology in confluent culture by 18^th^ day (Fig. 1a). Confluent culture of BMSCs was also obtained at 18^th^ day of culture. All type of cells (BMSC and DMSC) were scaled up further & characterized at 3X. Differentiation studies were carried out in cells from passages 3–8.

**Figure 2:**
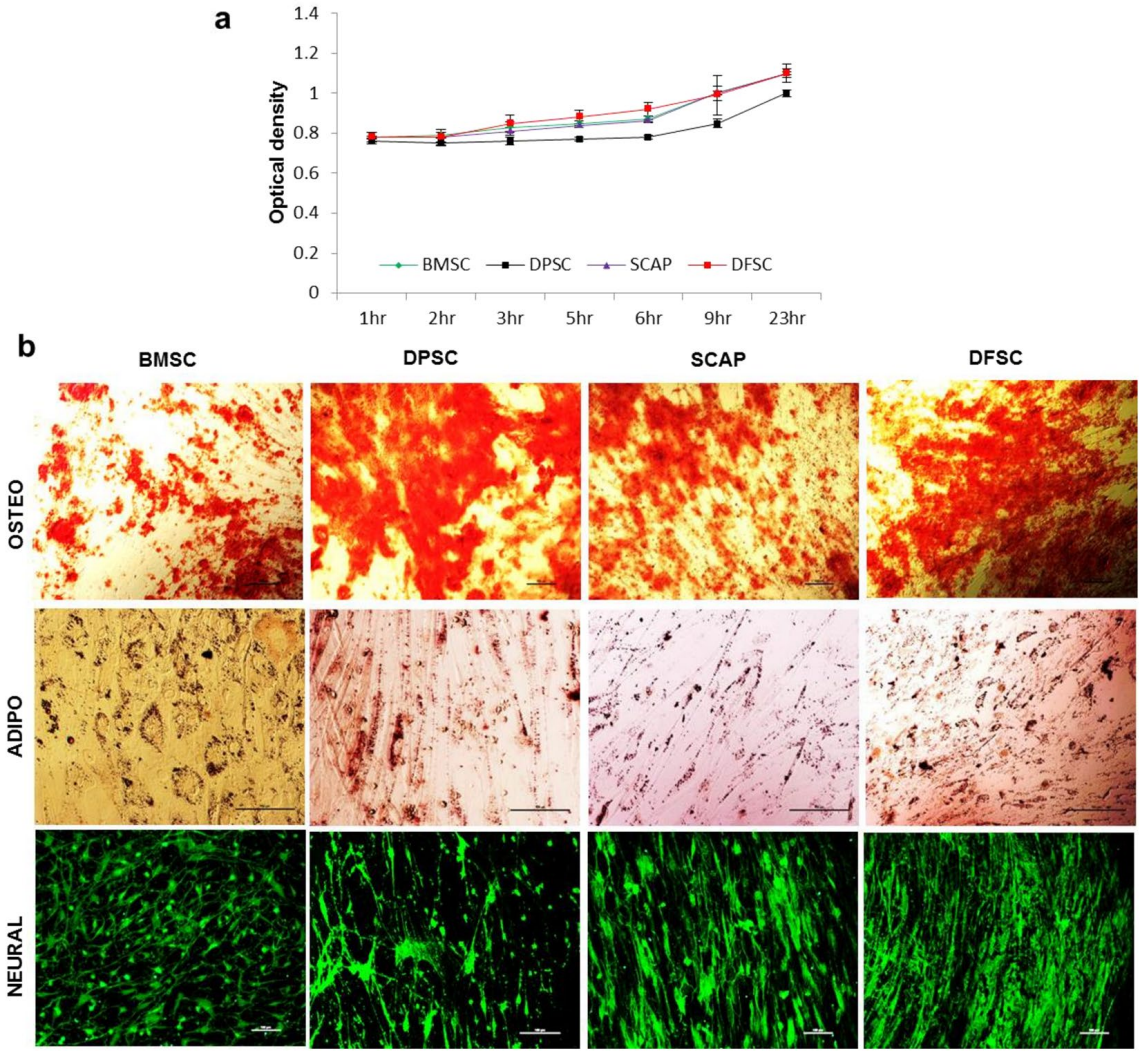
Proliferation and multipotency assessment of primary stem cells. (**a**) Cell proliferation analysis of BMSCs and DMSCs by EZB cell proliferation assay. (**b**) Multilineage differentiation of BMSCs and DMSCs into Osteocytes (evident by alizarin red staining), adipocytes (indicated by Oil Red O staining) and neural cells (showing immunofluorescence of Neurofilament antibody in differentiated cells). Scale bar-100 µm.

Stem cell characterization showed that all type of stem cells (BMSC, DMSCs) exhibited >90% positivity for stemness markers (CD90 and CD73) while and negligible expression (<5%) for CD34 and CD45 (Fig. 1b). However, expression of CD-105 was lower in DMSC with DPSC, SCAP and DFSC (~25–30%) as compared to BMSC (>90%). Analysis of proliferation status by EZB cell proliferation assay kit (Himedia) showed no difference in the proliferation potential of DMSCs as compared to BMSCs & showed almost similar proliferation potential as shown in Fig. 2a. Further characterization into functional multilineage differentiation capability of BMSCs and DMSCs was carried out by differentiating them into osteocytes which was confirmed by alizarin red staining, adipocytes as indicated by Oil Red O stain, and neural cells as conferred by neurofilament antibody immunofluorescence staining after neural differentiation (Fig. 2b).

### Assessment of Hepatic potential of BMSCs & DMSCs

**Figure 3:**
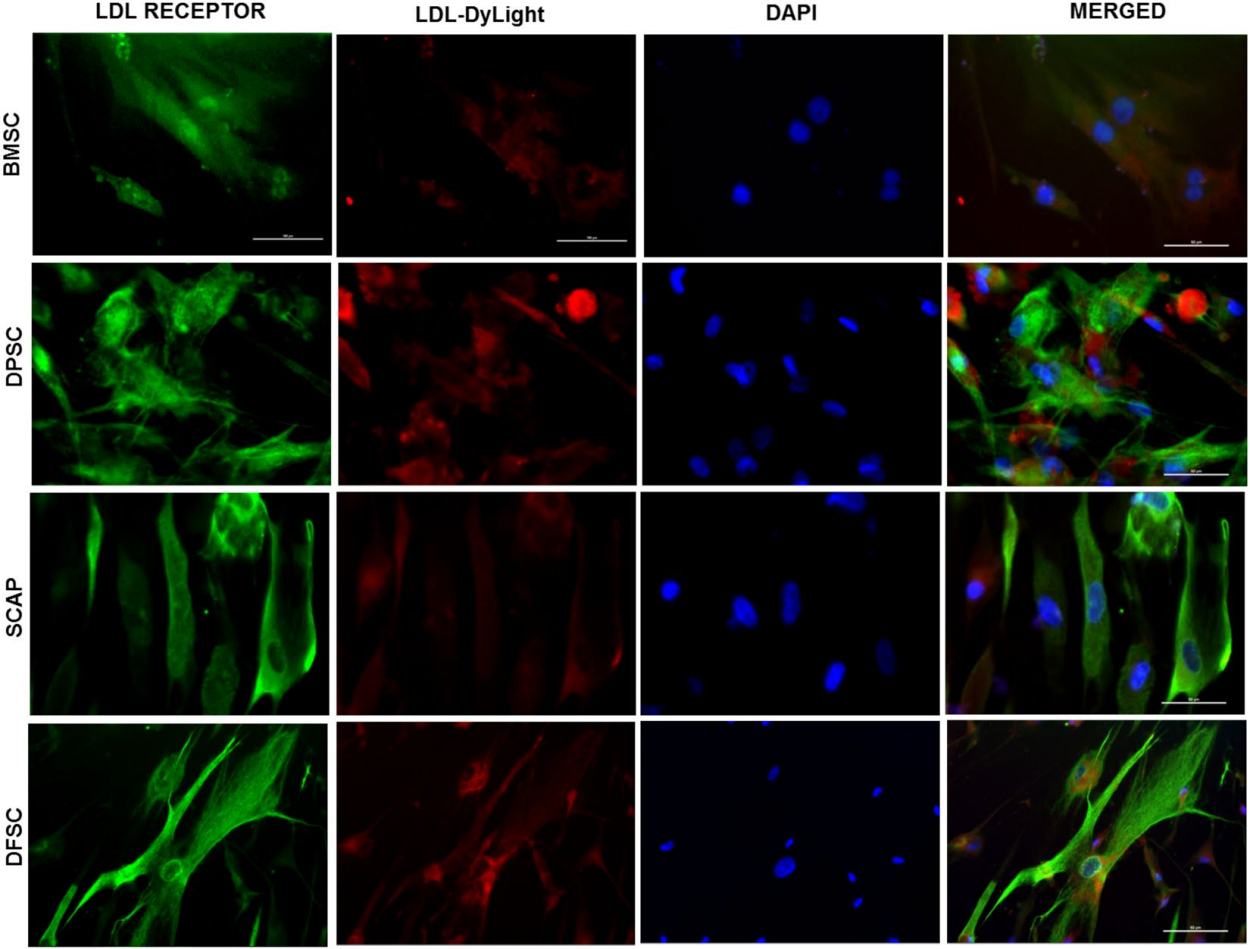
Functional characterization of hepatic differentiation in different stem cells. Immunofluorescence analysis of LDL receptor antibody distribution (green-FITC labeled) and LDL uptake (LDL DyLight fluorochrome-red, PE) in differentiated hepatocytes at 28^th^ day of hepatic differentiation. DAPI was used as nuclear stain. Scale bar-100 µm.

**Figure 4:**
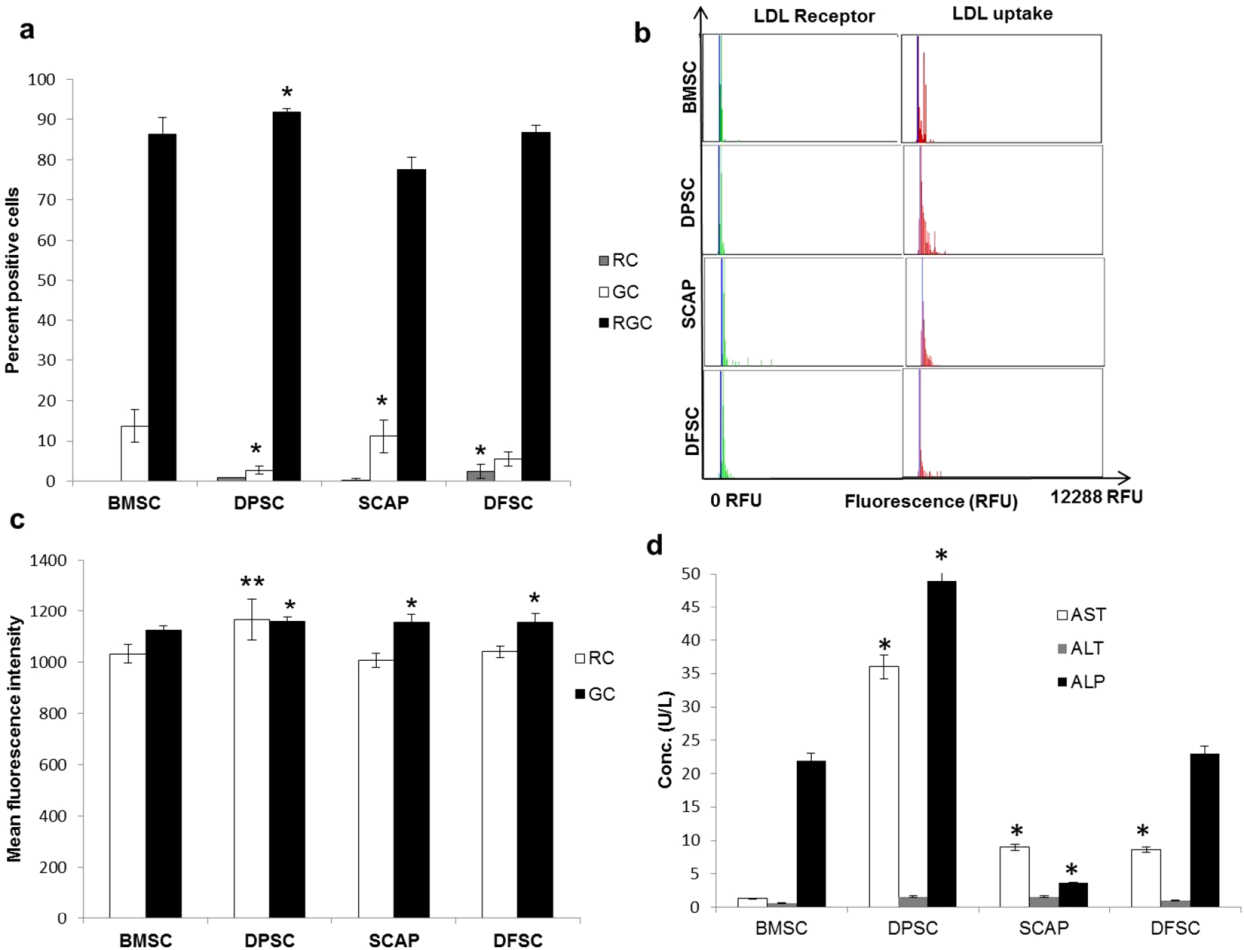
Comparison of LDL uptake in differentiated cells and assessment of liver parameters. (**a**) Bar diagram showing percentage positivity of LDL receptor (GC), LDL uptake (RC) and dual cells with expression of LDL receptor and uptake of LDL substrate (RGC). (**b**) Histograms showing relative fluorescence unit values for red and green fluorescence acquired by image cytometer. (**c**) Comparative analysis of average mean fluorescence intensity in red (RC) and green cells (GC). Fluorescence intensity of every positive cell was measured at single cell level and average were taken to drive the statistical significance. (**d**) Concentration distribution of major liver enzymes like AST, ALT and ALP in the BMSCs & DMSCs after hepatic differentiation. Statistical comparisons were made using BMSC as control. *P < 0.05, **P < 0.01, ***P < 0.01.

Hepatic differentiation potential of stem cells was assessed by functional uptake of low density lipoproteins by differentiated hepatocytes via functional LDL-hepatocyte assay kit, real time PCR analysis and evaluation of levels of certain liver parameters. Hepatic differentiation was carried out for a total period of 28 days. We observed that all cells acquired an oval morphology upon differentiation as compared to spindle shape morphology in undifferentiated state (Figure S1). Functionally differentiated hepatocytes are characterized by expression of LDL receptor and uptake of low density lipoproteins (LDL). A labeled LDL approach can make the real time analysis of LDL uptake simpler. So we characterized the differentiated hepatocytes from all four stem cell sources for expression of LDL receptor and by the degree of LDL uptake. Characterization of cells was done by LDL functional hepatocyte staining kit in which LDL antibody (green) bound to the LDL receptor present on the differentiated hepatocytes while functional LDL uptake was represented by LDL DyLight fluorochrome (red). All differentiated cells showed positive staining with LDL antibody as well as LDL DyLight fluorochrome as shown in Fig. 3. Quantitation of this staining was performed by two ways. First was the percentage of cells, positive for LDL receptor (green), LDL uptake (red) or both (green and red) which was recorded in image cytometer. Analysis of percentage positivity for both receptor and LDL uptake in the dual staining showed maximum positivity for DPSC as compared to BMSC, SCAP and DFSC for red and green cells (Fig. 4a,b). It may be noted that BMSC (13.7 ± 4.1%) and SCAP (22.2 ± 4%) showed higher number of green cells (with LDL receptor expression) as compared to other cells types, DPSC (7.2 ± 1%) and DFSC (10.7 ± 1.7) respectively. It reflected the presence of higher expression of LDL receptor, in differentiated BMSC and SCAP as compared to DPSC and DFSC. Analysis of average fluorescence intensity in this study showed that differentiated DPSC had higher MFI (1156 ± 19) as compared to differentiated BMSC (1123 ± 17.1) for both green and red cells respectively as shown in Fig. 4c. SCAP (1155 ± 29.5) and DFSC (1155 ± 33.5) also showed higher MFI for green cells as compared to BMSC. LDL uptake analysis also showed similar pattern as differentiated DPSC showed highest MFI for LDL uptake (1165 ± 80.9) as compared to differentiated BMSC (1030 ± 36.8) while there was no major difference in the MFI of red cells between BMSC, SCAP and DFSC.

**Figure 5:**
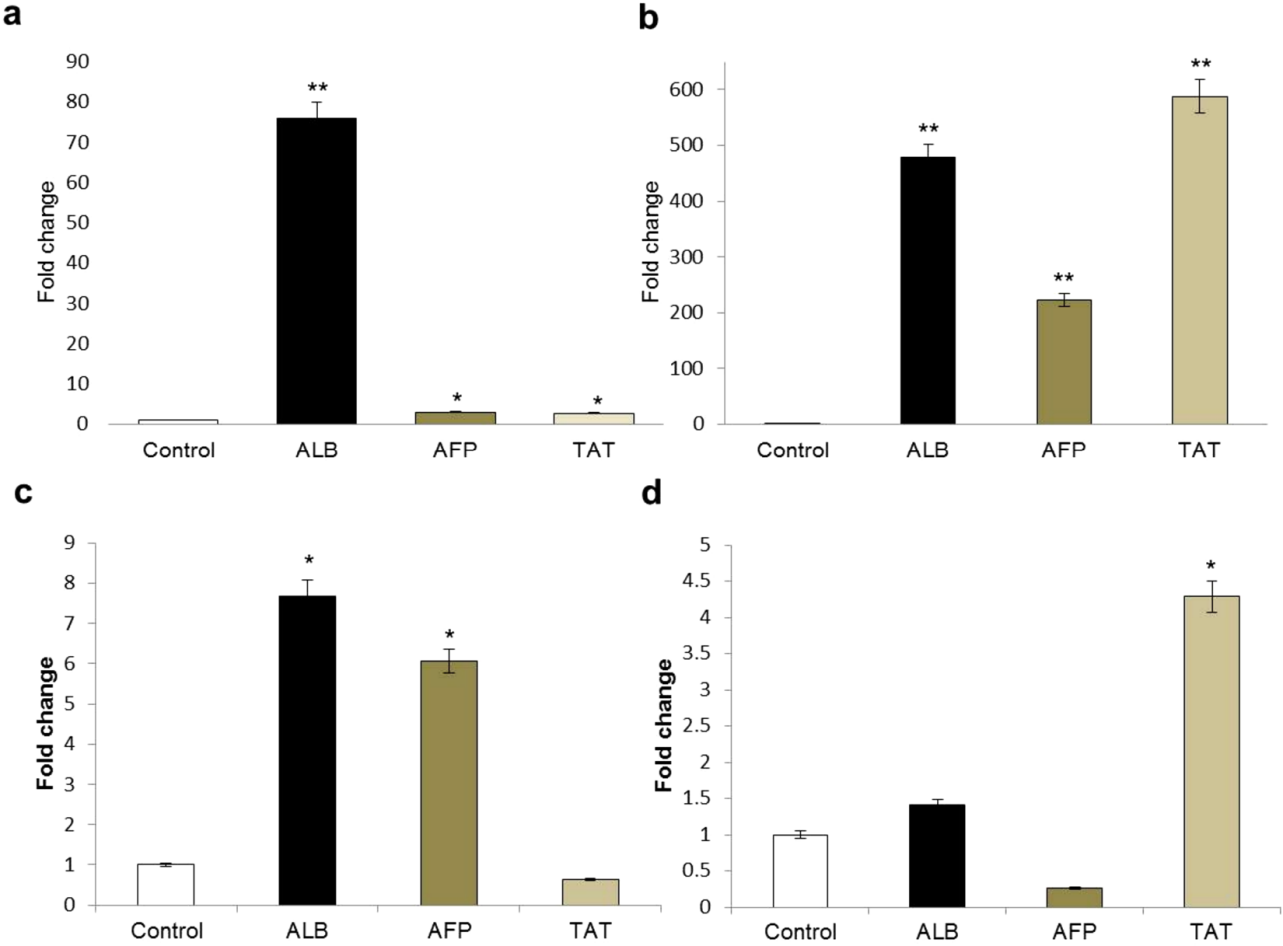
Real time PCR analysis for gene expression pertaining to hepatic lineage. (**a**) Bar diagram showing fold increase in expression of albumin (ALB), α-Fetoprotein (AFP) and Tyrosine amino Transferase (TAT) in differentiated BMSCs. (**b**) DPSCs. (**c**) SCAP, and (**d**). DFSCs respectively. Statistical comparisons were made with respective undifferentiated controls for each cell type. *P < 0.05, **P < 0.01, ***P < 0.01.

Albumin (ALB) and tyrosine amino transferase (TAT) are important markers for mature hepatocytes while α-fetoprotein (AFP) is an early hepatocyte marker. The sequence of gene primers used for real time is shown in Table S1. Expression of these markers in differentiated cells by real time PCR analysis revealed a significantly higher expression of ALB in differentiated DPSC as compared to BMSC while SCAP and DFSC showed minimal increase as shown in Fig. 5a,d. AFP expression was found to be highest in differentiated DPSC. TAT also showed a significantly higher expression in differentiated DPSC as compared to BMSC, SCAP and DFSC.

AST (Aspartate aminotransferase), ALT (alanine aminotransferase) and ALP (alkaline phosphatase) are important enzymes associated with liver function and of potential importance in depicting the differentiation of stem cells into functional hepatocytes. Measurements of these parameters were performed biochemically (Fig. 4d) to get an insight into functional efficacy of differentiated hepatocytes. It was observed that AST was significantly higher in differentiated DPSC (36 ± 20.5 U/L) as compared to differentiated SCAP (9 ± 5.2 U/L), DFSC (8.6 ± 5 U/L) or BMSC (1.3 ± 0.5 U/L). Similarly, ALP activity was highest in differentiated DPSC (49 ± 25.6 U/L) as compared to differentiated BMSC (22 ± 10.4 U/L), SCAP (3.6 ± 0.5 U/L) or DFSC (23 ± 5.1 U/L). Levels of ALT were also higher in differentiated DPSC and SCAP (1.6 ± 1.1 U/L in both) as compared to differentiated DFSC (0.6 U/L) or BMSC (0.6 ± 0.5 U/L).

**Figure 6:**
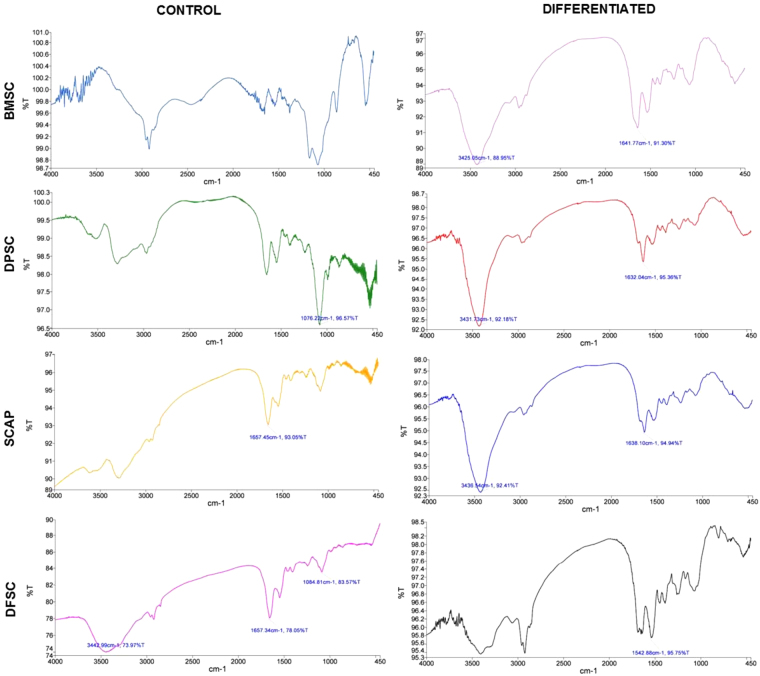
Fourier Transform Infrared spectroscopy for molecular signature investigation. Graphs showing the FTIR peak profile with unique wavenumber for each stem cell type (BMSCs & DMSCs) at control and differentiation level.

The molecular characterization of hepatic differentiation from four stem cell populations was carried out using Fourier Transform Infrared Spectroscopy (FTIR) and the peak profile for the same are shown in the Fig. 6 and Table 1. It may be noted that all differentiated stem cells showed a major peak at 3425–3436 cm^−1^ (except differentiated DFSC) and a secondary peak from 1542–1641 cm^−1^ as shown in Table 2.

**Table 1:** FTIR peak profile of mesenchymal stem cells (BMSC & DMSCs) after hepatic differentition.

Stem cell type	Peak wavenumber (cm^−1^)	Transmittance (%)	Inference	Reference
H-BMSC	3425	88.9	Stretching O-H asymmetric	31,32
	1641.1	91.3	Amide I band of protein and H-O-H deformation of water	
H-DPSC	3431.7	92.1	N-H stretching bands of mainly trans-ordered substructures	
	1632	95.3	Ring C-C stretch of phenyl, C=C uracyl, C=O	
H-SCAP	3436.5	92.4	Stretching O-H asymmetric	
	1638.1	94.9	C=C thymine, adenine, N-H guanine	
H-DFSC	1542.8	95.7	Amide II absorption (primarily an N-H bending coupled to a C-N stretching vibrational mode)

**Table 2:** List of the proteins present in the secretome of BMSCs and DMSCs with their specific function in context of hepatic lineage development and differentiation.

Sr. No.	Accession number	Protein ID	Name of protein	BMSC	DPSC	SCAP	DFSC	Hepatic lineage specific function
1	B7Z2B6	APC	Adenomatous polyposis coli protein	✓	×	✓	✓	Plays a role in hepatocyte growth factor (HGF)-induced cell migration
2	Q86TG7	PEG10	Retrotransposon-derived protein PEG10	✓	×	×	✓	Prevents apoptosis in hepatocellular carcinoma. May also have a role in cell growth promotion and hepatoma formation
3	P21439	ABCB4	Phosphatidylcholine translocator ABCB4	✓	×	✓	×	Mediates the translocation of phosphatidylcholine across the canalicular membrane of the hepatocyte. Export of ions and drugs from cytoplasm
4	Q0VD83	APOBR	Apolipoprotein B receptor	✓	×	×	×	Facilitate the fast uptake of Chylomicrons and VLDL
5	Q9UIR5	APOA	Apolipoprotein(A)	✓	×	✓	×	Involved in the lipid metabolism and also essential protein part in good cholesterol “HDL”
6	Q1HP67	LPA	Lipoprotein, Lp (A)	✓	×	×	×	Found at hepatocyte cell surface and plays a major role in hepatic regeneration
7	O75581	LRP6	LDL receptor-related protein 6	×	✓	×	×	Involved in regulation of canonical Wnt signalling pathway. Also control level of LDL and triglycerides in plasma
8	Q7Z4F1	LRP10	LDL receptor-related protein 10	×	✓	×	×	Facilitates the intake of APOE (lipoprotein)
9	O75197	LRP5	LDL receptor-related protein 5	×	✓	×	×	Involved in cholesterol metabolism by increasing the clearance of chylomicrons from hepatic system
10	O75096	LRP4	LDL receptor-related protein 4	×	✓	✓	×	Involved in the endocytosis of lipoproteins
11	Q14393	GAS6	Growth arrest-specific protein 6	×	✓	✓	✓	Promotes hepatic regeneration
12	H0YJ88	LRP1	ProLDL receptor-related protein 1	×	×	✓	×	Involved in the metabolism of lipoproteins and enhances chylomicron clearance
13	Q59EV4	LRP1B	Low density lipoprotein-related protein 1B	×	×	✓	×	-do-
14	Q14114	LRP8	LDL receptor-related protein 8	×	×	✓	×	Regulate lipid metabolism during early stage
15	O75074	LRP3	LDL receptor-related protein 3	×	×	✓	×	Helps in intake of lipophilic substances
16	D3DQQ7	LRP4	LDL receptor-related protein 4, isoform CRA_a	×	×	✓	×	-do-
17	A3KPE2	APOC3	Apolipoprotein C-III	×	×	✓	×	Inhibits lipoprotein lipase, hepatic lipase & reduces the endorsement of chylomicrons by hepatocytes thus interrupting the catabolism of TG-rich particles
18	B6ZGT4	HNF4G	Hepatocyte nuclear factor 4 gamma	×	×	✓	×	Hepatocyte nuclear receptor
19	P13725	OSM	Oncostatin M	×	×	×	✓	Involved in the maturation of fetal hepatocytes, thereby promoting liver development and Regeneration
20	P08581	HGFR	Hepatocyte growth factor receptor	×	×	×	✓	Receptor for HGF

-LDL-Low density lipoprotein.

-TG-Triglycerides.

Based on all the above parameters, a score was assigned to each stem cell (BMSCs & DMSCs) for assessment of degree of differentiation in hepatic lineage and the score was found to be highest for DPSCs (please see Table S2).

### Identification of key hepatic lineage related proteins in stem cell secretome

**Figure 7:**
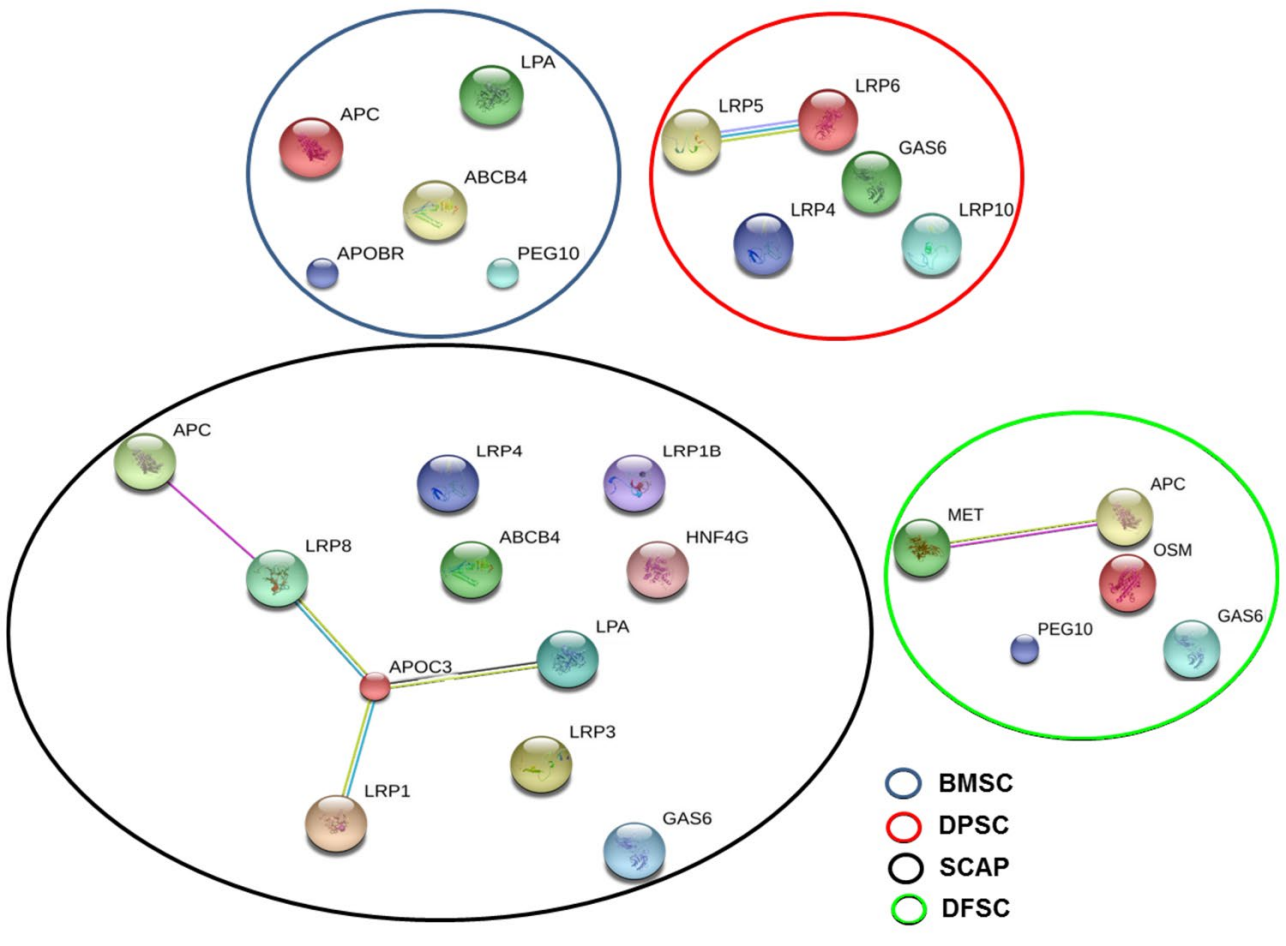
Interactome analysis of secretome proteins with relevance to hepatic lineage. Interaction analysis of different proteins pertaining to hepatic lineage in secretome of BMSC and DMSCs at baseline undifferentiated state using STRING software. Small nodes represent protein of unknown 3D structure while large nodes showed that 3D structure is known about the protein. Colored nodes represent the query proteins and edges represent protein-protein interaction. Green and red edges represent neighborhood proteins and fusion proteins.

The key proteins present in the secretome of different stem cells were identified by LC-MS/MS and we obtained a diverse protein profile pertaining to hepatic lineage development and differentiation. These involved hepatocyte growth factor receptor, oncostatin M, hepatocyte nuclear factor, growth arrest specific protein, LDL receptor family proteins etc. The detailed list of proteins associated with hepatic lineage alongwith their functions are provided in Table 1. A total of twenty proteins related to hepatic lineage were identified in secretome of four stem cell populations. Out of these, five proteins were identified in DFSC secretome which also included oncostatin M and hepatocyte growth factor receptor (the two main proteins used for in hepatic induction and maturation phase during *in vitro* culture). Twelve proteins were obtained in SCAP secretome while BMSC secretome showed six different proteins related to hepatic cell growth and export of drugs from hepatocytes. DPSC secretome showed five proteins one of which included Growth arrest specific protein 6 (GAS6) which is mainly associated with hepatic regeneration. Interactome analysis of these proteins by STRING bioinformatics software (Fig. 7) revealed an interaction between secretome proteins of DPSCs and SCAP while no interaction was observed between BMSC and DFSC secretome proteins. Further Reactome analysis revealed the involvement of six biological pathways in DPSC secretome which involved LRP5/LRP6 complex (Table 3). Reactome analysis in SCAP demonstrated the presence of two pathways in SCAP secretome involving APOC3, LRP1 and LRP8.

**Table 3:** Reactome giving interaction record of different proteins found in stem cell secretome and their association with different pathways.

Cell type	Associated Pathway	Proteins members present in secretome
BMSC	—	—
DPSC	Biochemical Reaction: GSK3beta mediated phosphorylation of cytoplasmic domain of LRP5/6	LRP6 and LRP5
	Biochemical Reaction: frog CK1gamma phosphorylates LRP5/6	-do-
	*Biochemical Reaction: CSNKI mediated phosphorylation of of cytoplasmic domain of LRP5/6	-do-
	Catalysis: phosphorylation of LRP5/6 cytoplasmic domain by membrane-associated GSK3beta	-do-
	Complex: WNT:FZD:p5S/T-LRP5/6:DVL:AXIN:GSK3B	-do-
	Complex: WNT:FZD:p10S/T LRP5/6:DVL:AXIN:GSK3B	-do-
	Catalysis: of Biochemical reaction pathway no. 3*	-do-
	Pathway: Transport AXIN to membrane by dissociating the destruction complex	-do-
SCAP	Catalysis: LRPs transport extracellular CR:atREs:HSPG:apoE to cytosol	APOC3, LRP1 and LRP8
	Pathway: Retinoid metabolism and transport	-do-
DFSC	—	—

## Discussion

The advantage of using dental stem cells as a source of cells for clinical research lies in their ease of isolation, no ethical constrains since they are derived from a biological waste, minimally invasive when compared to other stem cells like embryonic stem cells (ethical constraints), umbilical cord stem cells (lost, if missed at birth) and BMSCs (low cell number and highly invasive procedure). According to the minimal criteria defined by the International Society for Cellular Therapy, a stem cell should show adherence to the plastic adherence and characteristic expression of surface markers such as CD73, CD90, and CD105, they also display a negative expression of CD14, CD34, and CD45, and they should be capable of osteogenic, chondrogenic, and adipogenic differentiation^20,21^. Cells obtained according to our culture protocol from BMSCs and DMSCs were positive for mesenchymal markers and did not show presence of any contaminants from endothelial and hematopoietic lineage as confirmed by flow cytometry analysis. Multilineage differentiation potential of these cells into cellular derivatives of three germ layers further confirmed that the isolated population comprised of mainly mesenchymal stem cells.

Hepatic differentiation of stem cells includes induction followed by cell maturation. Stem cells can be made to differentiate into hepatocytes by various chemicals like hepatocyte growth factor (HGF), Oncostatin M (OSM), insulin, transferrin^22^. We employed a two-step strategy of HGF in first stage and hepatic maturation by OSM in the second stage. HGF is a potent mitogen for mature hepatocytes and is involved in proliferation and differentiation of oval cells in combination with other factors by a paracrine mechanism^23^. OSM induces the expression of hepatic genes like tyrosine amino transferase, glucose-6-phosphatase and accumulation of glycogen granules in developing hepatocytes and induce hepatic maturation by activating STAT3^24^. OSM also upregulate and initiate the secretion of albumin. Insulin has been reported to increase hepatic differentiation by activation of PI3/AKT pathway^25^. Hepatic differentiation is assessed based on some important parameters like enhanced expression of hepatic genes, uptake of low density lipoproteins (LDL), and by other biochemical parameters important for liver functions.

Increased expression of various genes like albumin, α-fetoprotein (AFP) and Tyrosine amino transferase (TAT) in differentiated cells showed presence of hepatocyte-like cells. Albumin (ALB) is the most abundant protein synthesized by hepatocytes. Fetal hepatocytes begin synthesizing ALB which increases in adult/mature hepatocytes making it an important marker of hepatocyte maturation^26^. α-fetoprotein is another early hepatocyte marker which is expressed at initial stages endodermal differentiation^26,27^. Contrary to ALB, its expression decreases in mature hepatocytes. Tyrosine amino transferase (TAT) is a marker for pre or postnatal hepatocyte differentiation as it is absent prior to birth but rapidly increases its levels during neonatal development^26,28^. A significantly higher expression of mature hepatocyte markers ALB and TAT as compared to AFP (early hepatocyte marker) in differentiated DPSCs reflected a more potent and mature hepatocyte formation in case of DPSCs in comparison to other stem cells.

Uptake of low density lipoproteins (LDL) is an important assay which is often used to demonstrate the activity of hepatocyte like cells on differentiation. Deficiency of LDL receptor has been reported to be associated with diseases like familial hypercholesterolemia (FH)^29^. Presence of a higher percentage of functional hepatocytes (dual green and red cells) after differentiation of DPSCs reflected it’s superiority for hepatic differentiation in comparison to rest of the stem cells. A low percentage of only green and only red cells in this population again confirmed this point. Further validation of the same by higher MFI in hepatocytes from differentiated DPSC strongly supported the notion that DPSCs have an edge over others for deriving functional hepatocytes.

Alanine amino transferase (ALT), Aspartate amino transferase (AST) and alkaline phosphatase (ALP) are three important enzymes associated with liver function. ALT is expressed at a higher concentration in liver as compared to other tissues of body. AST is an important enzyme associated with liver activity and ALP is associated with an increased proliferation of hepatocytes^30^. Increased levels of AST, ALT and ALP during serum tests serve as important markers for release of these enzymes by ruptured membrane of hepatocytes. We obtained these enzymes in lysate of differentiated hepatocytes from all four types of stem cells. Increase in the levels of AST, ALT and ALP in differentiated hepatocytes derived from DPSC again reflected the better hepatogenic potential of these cells as compared to three other stem cell types.

Very few reports are available about molecular signature profiling of hepatic differentiation by FTIR analysis. Thumanu *et al*. have characterized the spectroscopic signatures of differentiated hepatocytes at early and late stage by using mouse embryonic stem cells (mESCs)^31^. Amide I and II (ranged from 1500–1700 cm^−1^) bands have been shown to be associated with hepatic differentiation with Amide I found to be more potent during later stages of hepatic differentiation while Amide II to be more dominant for initial endodermal induction (induction of hepatic differentiation). This group reported the predominance of Amide II band in initial stages of endodermal induction while Amide I band was found to be associated with presence of α-helix protein structure of albumin (1656 cm^−1^)^32^. Interestingly the group also showed a peak shift of 1627–1641 cm^−1^ from induction to maturation during hepatic differentiation which also reflects a shift from β sheet structure to α helix. In differentiated BMSCs the band envelope of Amide I was observed at peak with wave number 1641.1 cm^−1^ which arises mainly due to the stretching and bending type of vibrations in backbone of the protein. This peak is also a characteristic peak for urea (related to detoxifying function of hepatocytes). An Amide II band was observed during differentiation of DFSCs (1542.8 cm^−1^) which also arises due to similar kind of vibrations. α- helix is generally centered at ~1650 cm^−1^ and the bands corresponding to this position were present in differentiated BMSCs, DPSCs (1632 cm^−1^) and SCAP (1638.1 cm^−1^). This can be explained by increase in the proteins which are α-helical in nature like albumin (the major protein produced in differentiated hepatocytes) during hepatic differentiation.

As albumin is a mature hepatocyte marker, the real time PCR results showed maximum increase in ALB in DPSCs and BMSCs in our differentiation system which was in agreement with maturation peak of 1632 cm^−1^ and 1641 cm^−1^ in differentiated BMSCs and DPSCs respectively.

The secretory factors present in niche can effect a cell’s decision for a specific cell fate and these micro environmental cues may be extrinsic or intrinsic. However, the cascade of molecules which orchestrate these signals are still not very clear. An elevated expression of the secretory factors during differentiation might play a role in regulating the differentiation potential of stem cells by a paracrine or autocrine manner. Higher expression of the secretory protein, NPTX1 during neural differentiation of induced pluripotent stem cells (iPSCs), might explain the neural promotory effect of NPTX1 on iPSCs^33^.

In similar lines, we sought for the presence of potential hepatic lineage associated proteins in stem cell secretome of all four populations. The divergent behavior of these cells towards hepatic differentiation was revealed by LC-MS/MS analysis. We obtained twenty different proteins pertaining to hepatic lineage maintenance and difference in secretome of these four stem cells as shown in Table 2. Adenomatous polyposis coli (APC), expressed in BMSC, SCAP & DFSC secretome is an important protein related to hepatic regeneration. Mutations in APC have been shown to increase the threat for development of hepatoblastoma^34,35^. It acts as a regulator of Wnt signaling pathway by controlling the hepatoma formation and has been found to be associated with accelerated liver regeneration after partial hepatectomy by modulating Wnt signaling. It also acts in a synergistic way with hepatocyte growth factor (HGF) to increase cell migration^36^. Retrotransposon-derived protein paternally expressed 10 (PEG10) (expressed in BMSC & DFSC secretome) has been shown to increase cell survival and growth in hepatic cancer^37^. ABCB4 (in BMSC and SCAP secretome) and hepatocyte nuclear factor 4 (in SCAP secretome only) have been reported to control the transport of organic anions and drugs and hence aiding in liver regeneration^38^. Apolipoprotein C-III (APOC3) is a VLDL protein and abrogate the hepatic and lipoprotein lipase^39^. Presence of this protein only in SCAP secretome might be a suggestive of limited hepatic potential of SCAP in comparison to DPSCs. Growth arrest-specific protein 6 (GAS6) is another important protein which boost hepatic regeneration in combination with its receptor Axl by increasing proliferation in hepatocytes. In an interesting study addition of GAS6 in culture was found to enhance the number of WB-F344 cells (hepatocyte precursor cell line)^40^. Presence of GAS6 in DMSC (DPSC, SCAP & DFSC) secretome and its absence in BMSC secretome might account for increased hepatic differentiation of these cells. Oncostatin M (OSM) (found in DFSC secretome) has been found to induce hepatic maturation by STAT3 signaling pathway^24^ and was also involved in termination of embryonic hematopoiesis in fetal liver^41^. Hepatocyte growth factor receptor (HGFR) (observed in DFSC secretome) acts as a receptor to HGF and has been associated with hepatic differentiation (initial commitment) and also in regulation of hepatic metabolism by stimulating glucose uptake^42^. In addition, presence of different LDL receptor proteins (LRP) in secretome of different stem cells reflected the role of stem cell secretome proteins in controlling lipid metabolism and transport which ultimately determines the activity of differentiated hepatocytes. Presence of different signalling molecules pertaining to lipid transport and metabolism in the secretome proteins of DPSCs and SCAP is an important indicator of enhanced tendency of these stem cells towards hepatic lineage. Thus, LC-MS/MS analysis revealed that in addition to the intracellular genes and proteins, complex interplay of secretome proteins may also contribute to the increased hepatic differentiation potential of DPSCs as compared to other stem cells. Differences based on the tissue of origin in stem cells play an important role in regulating the micro-environment which ultimately determine a stem cell’s inherent tendency to differentiate towards a particular lineage^18^. The secretome from DMSCs provides microenvironmental cues for hepatocyte differentiation.

### Conclusion and perspective for future research

Our study provides strong evidence for expanding potential of dental tissue as a viable source of mesenchymal stem cells as compared to bone marrow in context of hepatic lineage and regeneration. This study also emphasizes the fact that proteins secreted by BMSCs and DMSCs in their milieu also contribute towards inherent hepatic differentiation of these stem cells. These secretory proteins can offer a viable alternative to replace stem cell therapy with “cell free” therapy and may be future hotspots for developing potential therapies for various debilitating diseases.

## Electronic supplementary material

Supplementary Information
