# Supplementary material for: Molecular spectrum of secretome regulates the relative hepatogenic potential of mesenchymal stem cells from bone marrow and dental tissue
Source: Sci Rep. 2017 Nov 8;7:15015. doi: 10.1038/s41598-017-14358-0 (PMC5678086; doi:10.1038/s41598-017-14358-0)
Supplement: Supplementary file 1 — Supplementary Information [file 41598_2017_14358_MOESM1_ESM.docx]

**Molecular spectrum of secretome regulates the relative hepatogenic potential of mesenchymal stem cells from bone marrow and dental tissue**

Ajay Kumar^1^, Vinod Kumar^2^, Vidya Rattan^3^, Vivekananda Jha^2^, Arnab Pal^4^, Shalmoli Bhattacharyya^1*^

1. Department of Biophysics, PGIMER, Chandigarh, India

2. Department of Nephrology, PGIMER, Chandigarh

3. Unit of Oral and Maxillofacial surgery, Oral health science centre, PGIMER, Chandigarh, India

4. Department of Biochemistry, PGIMER, Chandigarh, India

**^*^Correspondence address:**

Dr. Shalmoli Bhattacharyya, PhD, FISBT, MAMS, Additional Professor,

Department of Biophysics, PGIMER, Chandigarh, 160012, India,

Tel: 91-9876186816, Fax: 91-172-2744401, E-mail: shalmoli2007@yahoo.co.in


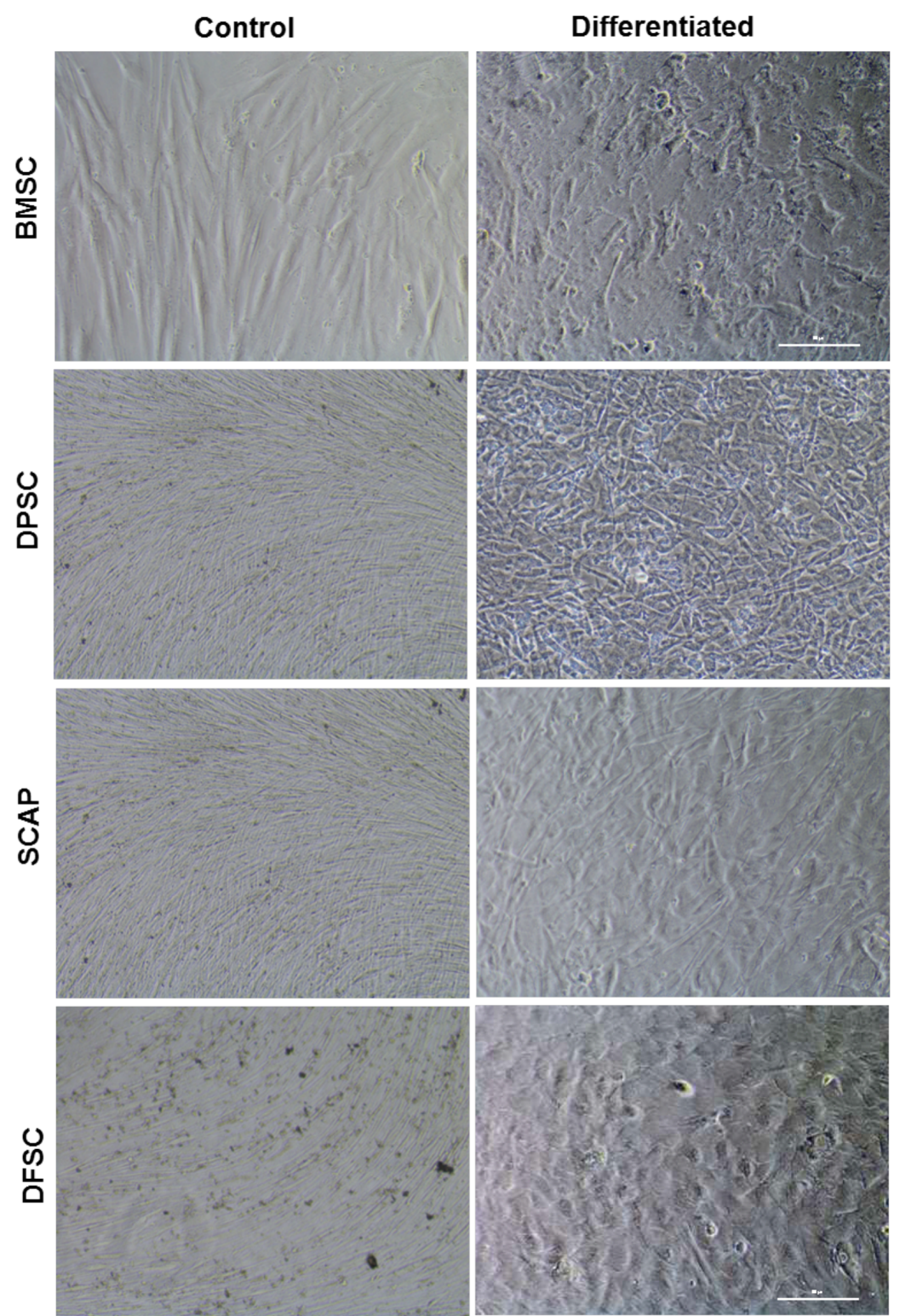


**Supplementary figure-S1.** **Morphology assessment of differentiated hepatocytes from four stem cells:** Phase contrast images of differentiated hepatocytes from BMSCs and DMSCs at 28^th^ day of hepatic differentiation. Scale bar-100µm.

**Table-S1**

| **PRIMER NAME** | **SEQUENCE** | **SIZE (bp)** | **Ref** |
| --- | --- | --- | --- |
| **Albumin** | **FWD:** AGCTGTTATGGATGATTTCGCAG  **REV:** CCTCGGCAAAGCAGGTCTC | 77 | 1 |
| **α-Fetoprotein** | **FWD:** AGCAGCTTGGTGGTGGATGA  **REV:** CCTGAGCTTGGCACAGATCCT | 88 | 1 |
| **Tyrosine Amino Transferase** | **FWD:** CCACACCCACACTCAGATCCT  **REV:** ATTAGTGAGTCACTCTAGCAGCGC | 76 | 1 |

**Supplementary table-S1.** Hepatic primers used for real time PCR BMSCs and DMSCs.

**Table-S2**

| **Hepatic Parameter** | **BMSC** | **DPSC** | **SCAP** | **DFSC** |
| --- | --- | --- | --- | --- |
| **LDL uptake** | 1 | 4 | 2 | 3 |
| **Real time PCR** | 2 | 4 | 1 | 3 |
| **Liver function test** | 2 | 4 | 2 | 3 |

**Supplementary table-S2.** Hepatic differentiation score of BMSCs, DPSC, SCAP and DFSCs based on various parameters used during the study.

**Reference:**

1. Józefczuk, J., Differentiation of human Embryonic Stem Cells into hepatocytes as a tool to analyse dynamic regulatory events during hepatogenesis in vitro. *Inaugural dissertation*. Freie University, 41, (2009).
